# A Nanoparticle-Based Model System for the Study of Heterogeneous Nucleation Phenomena

**DOI:** 10.1021/acs.langmuir.2c03034

**Published:** 2023-03-02

**Authors:** Ann-Kathrin Göppert, Guillermo González-Rubio, Simon Schnitzlein, Helmut Cölfen

**Affiliations:** Physical Chemistry, Department of Chemistry, University of Konstanz, Universitätsstraße 10, D-78465 Konstanz, Germany

## Abstract

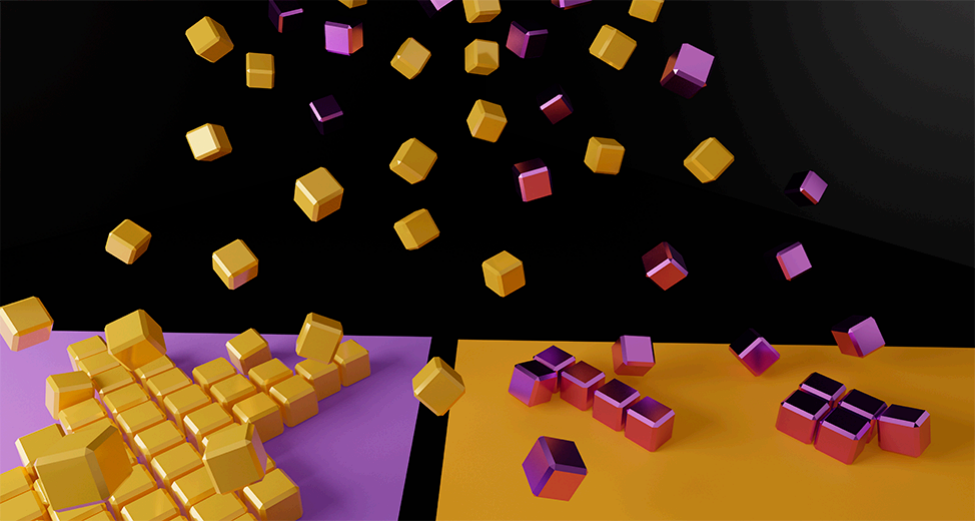

Heterogeneous nucleation processes are involved in many important phenomena in nature, including devastating human diseases caused by amyloid structures or the harmful frost formed on fruits. However, understanding them is challenging due to the difficulties of characterizing the initial stages of the process occurring at the interface between the nucleation medium and the substrate surfaces. This work implements a model system based on gold nanoparticles to investigate the effect of particle surface chemistry and substrate properties on heterogeneous nucleation processes. Using widely available techniques such as UV–vis–NIR spectroscopy and light microscopy, gold nanoparticle-based superstructure formation was studied in the presence of substrates with different hydrophilicity and electrostatic charges. The results were evaluated on grounds of classical nucleation theory (CNT) to reveal kinetic and thermodynamic contributions of the heterogeneous nucleation process. In contrast to nucleation from ions, the kinetic contributions toward nucleation turned out to be larger than the thermodynamic contributions for the nanoparticle building blocks. Electrostatic interactions between substrates and nanoparticles with opposite charges were crucial to enhancing the nucleation rates and decreasing the nucleation barrier of superstructure formation. Thereby, the described strategy is demonstrated advantageous for characterizing physicochemical aspects of heterogeneous nucleation processes in a simple and accessible manner, which could be potentially explored to study more complex nucleation phenomena.

## Introduction

Nearly 100 years after the classical nucleation theory was proposed to explain nucleation phenomena, significant knowledge gaps remain.^1−8^ From the formation of snowflakes and amyloid fibrils to the growth of mineral scales in pipelines, gaining insights into nucleation processes can help us to understand countless processes occurring in nature.^9−12^ Eventually, it is expected to deliver optimized methods for the fabrication of materials, preventing certain human diseases, or triggering rainfall.^13−15^ In general, heterogeneous nucleation events, where the new phase forms stimulated by surfaces, participate in a larger number of processes owing to its lowered activation barrier compared with homogeneous nucleation (the new phase forms uniformly throughout the parent phase).^16−19^ However, the nucleation phenomenon promoted by surfaces is generally more complex and less understood. This fact can be partially explained by a lack of analytical methods with sufficient spatial and temporal resolution to characterize the surface-assisted nucleation of critical nascent germs, which possess dimensions in the nanometer range.^17,19−22^

In this scenario, the use of colloidal particles has emerged as an attractive approach to investigate heterogeneous nucleation phenomena.^18,23−28^ As model systems, particles offer several advantages, such as larger dimensions and slower diffusion rates than molecules or atoms. For these reasons, nucleation events can be more easily investigated in colloidal systems using widely available techniques such as optical microscopy.^29−32^ For instance, the formation of gold nanocrystal-based structures can be monitored using UV–vis–NIR spectroscopy due to the changes in their optical properties during the nanocrystal assembly.^33−35^ Moreover, in colloidal building blocks, the interparticle potential can be tuned by rational control of the morphology, composition, and surface chemistry of colloidal particles, thereby simplifying comparison with experimental results, simulation, and theory.^27,30,36−40^ It is worth noting that, like atoms and molecules, they possess similar abilities to self-assemble into large crystals, such as superstructures or superlattices and mesocrystals (assemblies of faceted colloidal crystalline particles with equal crystallographic orientation).^36,41−47^ However, as in the case of atomic and molecular systems, the nucleation of mesocrystals and superstructures is not yet completely understood, complicating the development of optimized methods for their fabrication with desired properties.

In general, the critical size of a nucleus can typically be tuned by controlling the interfacial energy.^1,17,19^ For instance, it becomes smaller when the interfacial energy decreases, thereby favoring nucleation processes. This fact implies that nucleation can be tuned by adapting the interfacial energy of nanoparticle–substrate systems.^32^ In this scenario, the interfacial energy might be reduced by minimizing lattice strain (since the substrate’s structure matches a particular plane of the nucleating phase) or by allowing strong bonding to the nucleus.^48,49^ For example, surfaces derivatized with different functional groups can direct calcite crystal growth into specific orientations due to their impact on the nucleation plane.^50−53^ In colloidal systems, particle–particle interactions (hard-sphere, electrostatic, magnetic, and van der Waals interaction) critically determine their self-assembly and nucleation events.^36,45,54^ The interplay between colloidal forces, nanoparticle morphology, and substrate nature eventually provides control over the heterogeneous nucleation process.

The research described herein aims to develop a colloidal-based model system to study heterogeneous nucleation phenomena following our earlier work describing the influence of particle anisotropy onto heterogeneous nucleation.^55^ Here, we have specifically focused on the effect of substrate properties on the nucleation of superstructures formed by cetyltrimethylammonium bromide(CTAB)-stabilized gold nanocubes (Au NCs@CTAB). The charge and hydrophobicity of quartz and mica substrates were modified through derivatization with organosilanes bearing different functional groups, and their impact on the nucleation of gold superstructures was investigated via a combination of UV–vis–NIR spectroscopy and light microscopy. This strategy was also successfully applied to investigate the role of nanoparticle surface chemistry by replacing CTAB with poly(acrylic acid).

## Materials and Methods

All chemicals were purchased and used without further purification. Propan-2-ol (C_3_H_8_O, ≥99.5%), ethanol (EtOH, ≥99.8%), methanol (MeOH, ≥99.5%), acetone (C_3_H_6_O, ≥99.5%), and hydrochloric acid (HCl, 37.0%) were purchased from VWR. Nitric acid (HNO_3_, ≥65%), sodium hydroxide pellets (NaOH, ≥98.0%), sodium borohydride (NaBH_4_, ≥97%), and l(+)-ascorbic acid (AA, ≥99%,) were purchased from Carl Roth. Hydrogen tetrachloroaurate trihydrate (HAuCl_4_·3H_2_O, ≥99.9%), hexadecyltrimethylammonium bromide (CTAB ≥99%), cetyltrimethylammonium chloride solution (CTAC, 25 wt % in H_2_O), hexadecylpyridinium chloride monohydrate (CPC, 99.0–102.0%), (3-aminopropyl)triethoxysilane, *N*,*N*-diisopropylethylamine (DIPEA), and poly(sodium 4-styrenesulfonate, *M*_w_ of 70000 g/mol) were purchased from Sigma-Aldrich. 3-(Triethoxysilyl)propylsuccinic anhydride, 3-acetoxypropyltrimethoxysilane, *n*-butyltriethoxysilane, and heptadecafluoro-1,1,2,2-tetrahydrodecyl)triethoxysilane were purchased from abcr. (α-Thiol, ω-bromo)-terminated poly(acrylic acid) (PAA-SH, *M*_w_ of 8500 g/mol) was purchased from Polymer Source Inc. Mica sheets were obtained from Micro to Nano with different shapes and a thickness of 0.15 to 0.21 mm and the highest grade V-1 quality. Double-polished Si wafers (orientation [100] of 5 mm length and 7 mm width) from Siegert Wafer were used for ellipsometry measurements. Milli-Q water (18.2 MΩ·cm) was used in all experiments.

### Substrate Derivatization

The surface derivatization was adapted from Crampton et al.^56^ and performed using a vapor phase diffusion under a protective gas atmosphere. Different materials have been used as surfaces: mica for the nucleation experiments under the light microscope, Si wafers for the ellipsometry measurements, and quartz cuvettes for kinetic measurements in the UV–vis–NIR spectrometer. Before derivatization, the different surfaces were cleaned. Mica surfaces were freshly cleaved using cello tape. The Si wafers were cleaned in an ultrasonic bath for 10 min with propan-2-ol, 10 min with ethanol, and 10 min with acetone. The quartz cuvettes were cleaned in *aqua regia* for 1 h and afterward cleaned thoroughly with water. The different surfaces were treated with an oxygen plasma for 10 min at 100% power (80 W) to generate free hydroxyl groups. A 4 L desiccator was evacuated two times and flooded with nitrogen. The substrates were placed in the desiccator, followed by evacuation and flooding with nitrogen for a third time to reach a humidity under 25%. Then, 60 μL of the corresponding liquid silane and 20 μL of 1,1-diisopropylethylamine were placed in the desiccator in separate beakers. The desiccator was evacuated for 30 s, sealed afterward, and left for different timespans (depending on the used silane) at RT. Afterward, the derivatized surfaces were treated for 2 h at 150 °C under ambient conditions to ensure the homogeneous distribution of the siloxanes on the surface and a complete cross-linking of the siloxane network. The different used silanes and their respective coating conditions are shown in Tabel S1. In the case of sulfonate derivatization, the (3-aminopropyl)thriethoxysilane derivatized surfaces were incubated in an aqueous solution of 1 wt % polystyrenesulfonate for 1 h, cleaned thoroughly with water, and dried at 50 °C. For the derivatization with carboxylic groups, the 3-(triethoxysilyl)propyl-succinic anhydride derivatized substrates were deprotected in water for 90 min at 50 °C and afterward dried at 50 °C in a drying cabinet. For the derivatization with hydroxyl groups, the 3-acetoxypropyltrimethoxysilane derivatized surfaces were deprotected by incubation in a 0.1–0.2 N sodium hydroxide solution of dichloromethane and methanol (9:1) overnight. Then, substrates were cleaned thoroughly with water and dried at 50 °C in a drying cabinet. All derivatized surfaces were used in a period of 1 to 2 days after the derivatization.

### Au NC Synthesis and Functionalization

Au NCs stabilized with hexadecylpyridinium chloride (Au NC@CPC) were synthesized in a three-step seed-mediated approach published by Kirner et al.,^57^ and CPC was exchanged with CTAB as follows: 50 mL of a 0.17 mM Au NC@CPC solution was centrifuged at 6000 rpm for 1 h. The supernatant was discarded, and the precipitated Au NC@CPC were transferred to a micro test tube. The concentrated nanoparticles were redispersed with a 2 mM CTAB aqueous solution and centrifuged at 6000 rpm for 20 min. This step was repeated twice. Finally, the concentrated nanoparticle sediment volume was determined to know the current concentration of the Au NC@CTAB. For the Au NC functionalization with PAA-SH (Au NC@PAA), 1 μL of concentrated CTAB-functionalized gold nanoparticles was diluted in 300 μL of water and added dropwise into a PAA-SH aqueous solution (14.3 mg/mL). To remove the excess of PAA-SH, the nanoparticles were centrifuged in micro test tubes for 5 min at 4000 rpm. The supernatant was discarded, and the precipitated Au NC@PAA nanoparticles were redispersed in 1 mL of water and centrifuged for 30 min at 4000 rpm. Finally, the supernatant was discarded, and the concentrated Au NC@PAA nanoparticles could be used for further experiments.

### Nucleation Experiments with Au NC@CTAB

To initiate the nucleation process, the Au NC@PAA nanoparticles were destabilized following a modified protocol published in a previous paper.^35^ Briefly, a certain volume of the concentrated AuNC@CTAB solution was added into an aqueous 0.02 mM CTAB solution to reach the desired AuNC@CTAB concentration (the stability of the nanoparticles and the concentration were checked with UV–vis–NIR spectroscopy). Then, ethanol was added to a final concentration varying from 12 to 38 vol % depending on the experiment (Table S2). The aggregation process was stopped at any time by readjusting the CTAB concentration to 2 mM. To analyze the structures formed on the mica surfaces, they were washed thoroughly with water and dried with a nitrogen stream at RT.

### Instrumentation

*Plasma cleaning*: a Miniflecto from PlasmaTechnology equipped with a 20–50 kHz, 80 W generator was used. *Contact angles (θ) and free surface energies*: the drop shape analyzer DSA25 and the software Advance from Krüss were used. Contact angles larger than 10° were fitted with the Ellipse (Tangent-1) model, while contact angles smaller than 10° were fitted with the Circle model. The free surface energies were calculated with the contact angles from water and diiodomethane over the Owens, Wendt, Rabel, and Kaelble (OWRK) model. *Dynamic light scattering*: measurements were performed on a Zetasizer Nano ZSP (Malvern Instrument, Malvern, UK) using a He/Ne laser (λ = 633 nm). *Zeta potentials*: measurements were performed on a Malvern Instruments Zetasizer Nano-ZS Zen3600. Transmission electron images were recorded on a Zeiss Libra 120 microscope or on a JEOL JEM-2200FS microscope at an accelerating voltage of 120 kV. The samples were deposited on Quantifoil carbon-coated Cu 400 mesh grids. *UV–vis–NIR spectra*: measurements were performed with a Varian Cary 50 spectrometer in quartz cuvettes. The UV–vis–NIR measurements performed simultaneously with light microscopy were performed with a modular USB2000+ spectrometer from Ocean Optics equipped with a USB-DT miniature deuterium tungsten halogen lamp. *Light microscopy*: images were recorded with an AxioImager from Zeiss with an LD Epiplan 50*x*/0.50 HD DIC objective using transmitted light, bright field illumination, a condenser numerical aperture at 0.9, an Axiocam 506 bw as an imaging device, and an exposure time of 10 ms. Light microscope pictures were taken every 30 s during the experiments and processed with ImageJ and Fiji. A Trainable Weka Segmentation was applied, differentiating between nuclei on the surface, background, and moving aggregates in solution. The classifier results were given out, and the number of nuclei species was counted. A threshold (MaxEntropy) was applied, and the number of nuclei was counted using the analyze particles function (size = 10 – infinity pixel). The number of counted species of the first picture (only background, structure growth did not start yet) was subtracted from all following results. *Scanning electron microscopy*: images were recorded with a Gemini500 by Zeiss operating at 3 kV equipped with an Inlens and a SE detector for secondary and backscattered electrons. Samples were sputter-coated with a 2.5 nm gold or platinum layer, mounted on aluminum stubs, and attached to carbon conductive tabs.

### Au NC@CTAB Concentration

The optical density of the Au NC@CTAB suspension at 400 nm was first recorded, and assuming an extinction coefficient ε_*λ*_ of 2.685 L/mol·cm for 23 nm Au NCs,^4^ we then determined the Au(0) concentration via the Beer–Lambert law:^58^
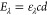
1where the optical path *d* of the utilized cuvettes was 1 cm. In the next step, the Au NC@CTAB concentration was retrieved by assuming that a 23 nm Au NC contains 717632 atoms (with a volume of 12176 nm^3^ and a lattice parameter of 0.4078 nm, a single AuNC contains 179408 unit cells constituted by 4 gold atoms).^59,60^ Then, with the determined Au(0) concentration and the number of gold atoms per Au NC it was possible to obtain the Au NC concentration.

### Supersaturation σ

The concentration of Au NCs in a saturated solution x* was determined for each AuNC–substrate system. Therefore, the Au(0) concentration after the destabilization of the nanocubes (when a steady state was reached) was determined with UV–vis–NIR spectroscopy, and the corresponding AuNC concentration was calculated. The resulting supersaturation for each destabilization experiment at an AuNC concentration *x* was calculated with eq 2:
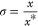
2

### Nucleation Rates *J*_n_

Au NC@CTAB colloids were destabilized in a fluorinated quartz cuvette in contact with sulfonate and carboxyl derivatized mica substrates through a small hole (ca. 6–8 nm diameter) introduced on one side of the cuvette where the mica substrate was placed. The cuvette was then filled with the reaction mixture; thereby, it was possible to observe the formation of Au NC@CTAB SSs on the mica substrates using an optical microscope. Moreover, we were also able to monitor the Au NC concentration during the duration of the heterogeneous nucleation experiment via UV–vis–NIR spectroscopy (i.e., utilizing a portable UV–vis–NIR spectrometer and white light source). Under the utilized experimental conditions, the formation of Au NC@CTAB SSs was only observed on the mica substrates, thereby enabling a constant supersaturation during the experiment. The number of counted structures increased linearly with time, indicating steady-state nucleation. The slope of the linear fit for this curve provides the nucleation rate.

## Results and Discussion

### Homogeneous Nucleation

Prior to investigating the heterogeneous nucleation of superstructures constituted by Au NCs, we first focused our attention on their self-assembly in solution. Thereby, we aimed to define optimal conditions to initiate the (homogeneous) nucleation for differently derivatized substrates. In this context, we synthesized 23 nm Au NCs (see the Materials and Methods section for more details and Figure S1) using CTAB as a shape-directing and stabilizing agent.^57^ The use of the cationic surfactant CTAB ensures high colloidal stability of the synthesized Au NC@CTAB colloids due to electrostatic repulsion (ζ potential of 15.8 mV for Au NC@CTAB, Figure S1).

**Figure 1:**
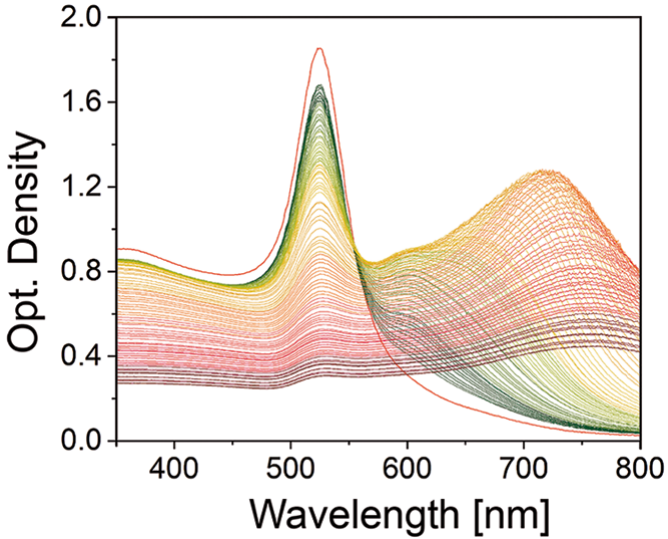
Homogeneous nucleation of Au NC@CTAB SSs. UV–vis–NIR spectra showing the LSPRs time evolution (for 4 h) of Au NC@CTAB during the homogeneous nucleation process of Au NC@CTAB-based superstructures

To initiate homogeneous nucleation of Au NC@CTAB superstructures (Au NC@CTAB SSs), we should first determine the optimal route to induce nanoparticle self-assembly meaning to weaken the electrostatic repulsive interaction responsible for maintaining the Au NCs in a dispersed state.^61^ For Au NC@CTAB, a controlled destabilization was achieved by adding ethanol (up to 38% v/v) due to removal of CTAB from the nanocrystal surface and subsequent decreases of repulsive interactions.^62^ Then, the homogeneous nucleation process was monitored according to the changes of the localized surface plasmon resonance (LSPR) band of Au NC@CTAB with UV–vis–NIR spectroscopy.^34^ When plasmonic Au nanoparticles self-assemble, new LSPR modes emerge due to the coupling of their LSPRs (Figure 1). The magnitude of such changes depends on the distance between the particles, the aggregation number, and the morphology of the assembled structure.^34,63^

In our case, 23 nm Au NCs display a narrow LSPR band centered at 523 nm that, upon addition of ethanol, slowly decreases in intensity as a result of the Au NC@CTAB aggregation (Figure 1). At the same time, new plasmon bands emerge at wavelengths above 600 nm, ascribed to the assembled Au NC@CTAB (i.e., the Au NC@CTAB SS nuclei). It is worth mentioning that the surface of the quartz cuvette employed to monitor the Au NC@CTAB destabilization process may influence the nucleation (it is practically not possible to work in the absence of surfaces or interfaces). To minimize such an issue, the cuvette surface was derivatized with a fluorinated organosilane to reduce interactions between the cuvette surface and Au NC@CTAB (see the Materials and Methods section and Table S1). Under such conditions, only the addition of large amounts of ethanol can trigger Au NC@CTAB SS formation via homogeneous nucleation. In this scenario, it is also important to point out that an air–water interface exists during the described homogeneous nucleation experiments, potentially impacting the homogeneous nucleation process. However, we did not observe the accumulation of Au NC@CTAB at such an interface (typically recognized due to the formation of a golden layer), which suggests that the air–water interface does not participate in the investigated nucleation phenomenon.^64,65^

Finally, to quench the aggregation process of Au NC@CTAB, an excess of CTAB can be added. Such a control over the nucleation process will be convenient for the heterogeneous nucleation investigations discussed in the following section.

### Heterogeneous Nucleation

**Figure 2:**
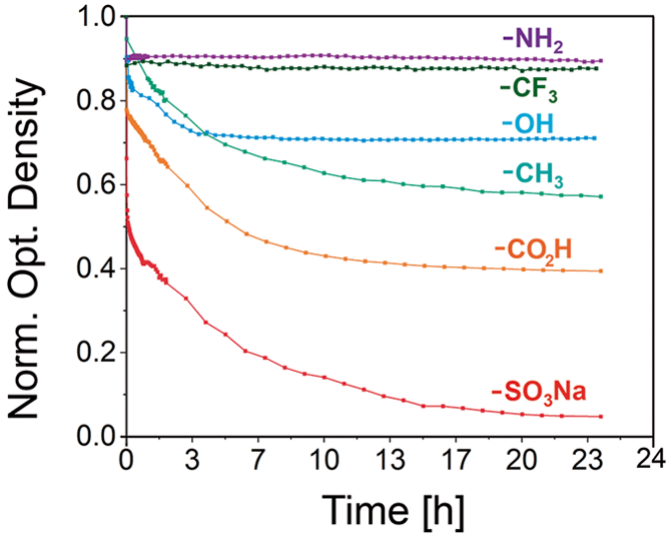
Heterogeneous nucleation of Au NC@CTAB SSs inside functionalized quartz cuvettes. Time evolution of the extinction of Au NC@CTAB at 523 nm during the heterogeneous nucleation of superstructures inside quartz cuvettes derivatized with −CO_2_H, −SO_3_Na, −NH_2_, −OH, −CH_3_, and −CF_3_.

Once the optimal conditions for homogeneous nucleation of Au NC@CTAB SSs were successfully established, we focused on their heterogeneous nucleation and the effect of using substrates with different surface chemistry. To get preliminary insight into it, we used quartz cuvettes, owing to the possibility of tuning its surface behavior with different organosilanes and tracking the aggregation kinetics using UV–vis–NIR spectroscopy. Quartz cuvettes were derivatized with carboxylic (−CO_2_H), sulfonate (−SO_3_Na), amine (−NH_2_), hydroxyl (−OH), and apolar (−CH_3_, −CF_3_) groups (see the Materials and Methods section for more details about the derivatization methodology and experimental conditions and Tables S1 and S2). Compared with the homogeneous nucleation experiments, we observed that a significantly lower amount of ethanol was required to trigger Au NC@CTAB SS nucleation (38% vs 15% v/v). This fact could potentially indicate a decrease in the nucleation energy barrier. Moreover, the derivatization nature of the quartz cuvette showed an evident influence on the nucleation process. For instance, Au NC@CTAB remained stable during the entire duration of the experiment (i.e., 24 h) in cuvettes functionalized with −NH_2_ and −CF_3_ (Figure 2). This could be explained by unfavorable interactions between −NH_2_ and −CF_3_ with Au NC@CTAB due to electrostatic repulsion (CTAB and −NH_2_ possess positive charges) and the repulsive interactions between fluorinated hydrophobic surfaces with very low van der Waals attraction and charges, respectively. In contrast, those cuvettes derivatized with −CO_2_H and −SO_3_Na enhanced the nucleation of Au NC@CTAB SSs, most probably due to favorable electrostatic interactions with the positively charged Au NC@CTAB. Finally, the −OH and −CH_3_ derivatized surface cuvettes’ influence was less significant than in the case of −CO_2_H and −SO_3_Na (Figure 2). The former may be explained by the weak negative charge of −OH moieties, while the latter could be attributed to van der Waals interactions between the cetyl chain of CTAB molecules and the −CH_3_ groups. In this context, obtaining quantitative information about the heterogeneous nucleation process rate might seem suitable. However, although the extinction at 523 nm corresponds solely to the Au NC@CTAB LSPR band maximum at the beginning of the nucleation process, the different Au NC@CTAB SSs species formed during the heterogeneous nucleation also contribute later to the extinction at 523 nm.^34,66^ This implies that it is highly challenging to determine the rate constants directly from the evolution of the extinction band (i.e., due to the overlapping contributions of different species), and a different strategy is required to access quantitative kinetic information.

To further support the reliability of the observed surface chemistry influence on the heterogeneous nucleation of Au NC@CTAB SSs, we reproduced the described nucleation experiments employing mica instead of quartz (Figures S2 and S3). Mica is atomically cleavable (i.e., smooth surface), which ensures minimal undesired nucleation processes potentially triggered by surface roughness.^67^ As in the experiments with quartz cuvettes, we derivatized mica substrates with −CO_2_H, −SO_3_Na, −NH_2_, −OH, −CH_3_, and −CF_3_ groups (see the Materials and Methods section). In this case, the experiments were performed in micro test tubes (three for each mica functionalization to ensure the acquisition of reliable data), and the nucleation processes were monitored via recording the UV–vis–NIR spectra of aliquots taken after 15 min and 24 h of nucleation experiment (Figures S2 and S3). It is important to note that we did not observe an influence of the micro test tube surfaces on the heterogeneous nucleation process, which allows us to properly investigate the effect of differently derivatized mica substrates on the formation of Au NC@CTAB SSs.

**Figure 3:**
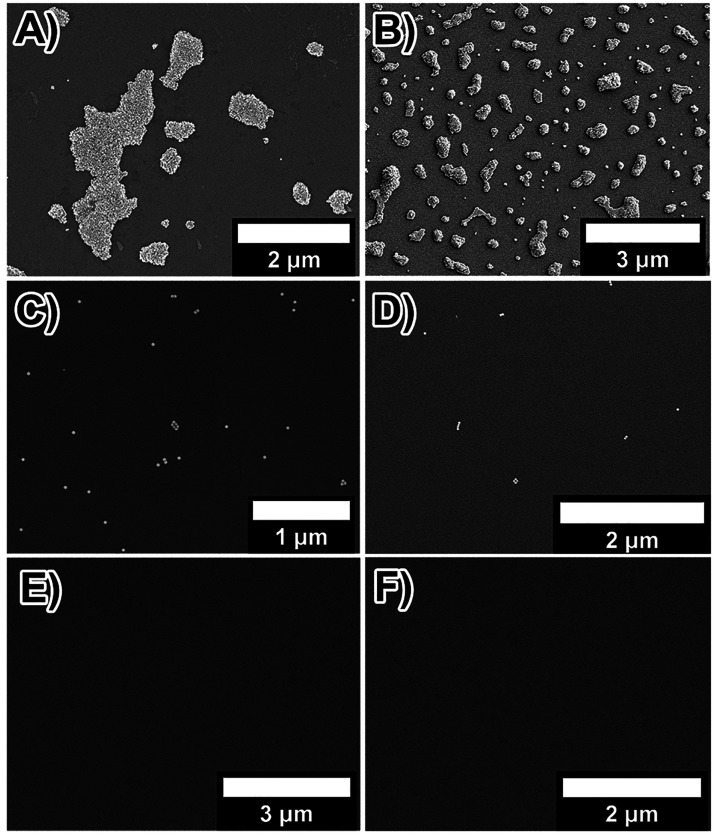
Heterogeneous nucleation of Au NC@CTAB SSs using derivatized mica substrates. SEM images of Au NC@CTAB SSs formed on mica substrates derivatized with −SO_3_Na (A), −CO_2_H (B), −OH (C), −CH_3_ (D), −NH_2_ (E), and −CF_3_ (F).

The results were consistent with the outcomes of the experiment performed in derivatized quartz cuvettes. For instance, mica substrates derivatized with −CO_2_H and −SO_3_Na showed a more prominent ability to induce heterogeneous nucleation of NC@CTAB SSs than those carrying −OH and −CH_3_ groups. No effect was exerted by mica substrates bearing −NH_2_ and −CF_3_ moieties. The preliminary information obtained with UV–vis–NIR spectroscopy was further confirmed through SEM characterization experiments. In this sense, −CO_2_H and −SO_3_Na (Figure 3A,B) derivatized substrates presented large NC@CTAB SSs, with dimensions ranging from 0.5 to 3 μm, while smaller superstructures (formed by a few Au NCs) and single Au NC were observed on the −OH and −CH_3_ derivatized mica substrates (Figure 3C,D). We did not notice structure formation for −NH_2_ and −CF_3_ derivatized substrates (Figure 3E,F).

### Nucleation Barrier

Once we successfully demonstrated the development of a nanoparticle-based model system to investigate heterogeneous nucleation processes, we focused on the study of associated physicochemical features. In this work, we have utilized the CNT to investigate the kinetic and thermodynamic aspects of the heterogeneous nucleation of Au NC@CTAB SSs, such as the nucleation rate *J*_*n*_ and the energy barrier, Δ*G*_*n*_ at the critical radius:^55,68,69^
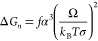
3where *f* is a numerical factor depending on the geometry of the nucleus, α is the effective interfacial energy between the nucleus and the medium, Ω is the volume per molecule of the solid phase, *k*_B_ is the Boltzmann constant, *T* is the temperature, and σ is the supersaturation. In this scenario, it is important to note that the CNT assumes that the properties of the involved species are bulk ones, continuum thermodynamics can be applied, and all associates of building units are spherical (this is taken into account via shape factors, *f*).

The nucleation rate *J*_*n*_ can be expressed as follows:
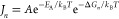
4where *A* is a prefactor (which is independent of σ) and *E*_A_ is the effective activation barrier (that typically accounts for kinetic barriers related to desolvation, diffusion, and rearrangement phenomena). By inserting (3) into (4), we arrive at
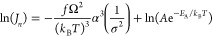
5where the right and left term account for the kinetic and thermodynamic contributions, respectively, Ω = 1.22 × 10^–23^ m^3^ as the volume of one Au NC with the edge length of 23 nm. Here we consider a spherical nucleus attached to a substrate, which geometry is defined by its contact angle. To give a possible range for the interfacial energies, these were calculated for contact angles of 60°, 90°, and 120° (the used form factors for each angle are given in Tables S3 and S4). Having a closer look at eq 5, it can be noticed that the logarithm of the nucleation rate ln(*J*_*n*_) is directly proportional to the inverse square of the supersaturation 1/σ^2^. By determining the magnitude of the slope, *s*, it is thus possible to calculate the interfacial energy, α, and eventually Δ*G*_*n*_:
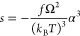
6Although the morphology of the nuclei formed on −CO_2_H and −SO_3_H derivatized mica surfaces deviate from the ideal spherical morphology assumed by CNT (Figure 3A,B), the conclusions on the nucleation rate should not be significantly impacted (i.e., *A*, *f*, and α will change, but not the experimentally obtained *s*). Moreover, due to the broad use of CNT to investigate nucleation phenomena, its utilization of CNT to evaluate the proposed system should allow us to compare the results for nanoparticles as building units with those of ions^48,69^ and gain insights into the feasibility of colloidal nanoparticles as a model system to investigate heterogeneous nucleation processes on the molecular scale.^55,70^ Notably, a nucleation theory taking the shape and interface of the building units and the nucleation surface into account seems to be not completely developed yet.

**Figure 4:**
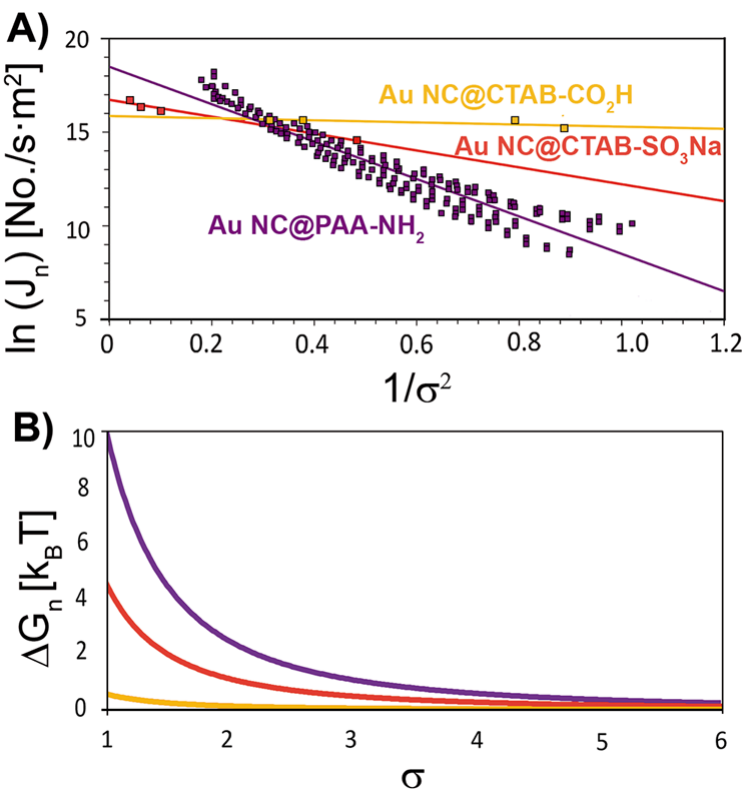
Nucleation rate and energy barrier. (A) Variation of the natural logarithm of the nucleation rates with the inverse square of the supersaturations and (B) plot of the evolution of the nucleation energy barrier with the supersaturation of Au NC@CTAB SSs using form factor of 8.38 and the retrieved interfacial energies corresponding to −SO_3_Na (red, α = 6) and −CO_2_H (orange α = 3) derivatized mica substrates and Au NC@PAA SSs formed on the −NH_2_ derivatized one (purple α = 8.2).

To carry out the proposed research investigation on the kinetic and thermodynamic aspects of the heterogeneous nucleation of Au NC@CTAB SSs, our characterization technique of choice was light microscopy, as it is highly advantageous to in-situ monitoring the growth of Au nanoparticle superstructures with sizes above 0.8 μm. Moreover, we combined it with UV–vis–NIR spectroscopy, following a procedure recently reported (see details in the Materials and Methods section).^70^ Thereby, we investigated the nucleation rates at different supersaturations for those substrates showing enhanced ability to promote heterogeneous nucleation of Au NC@CTAB SSs: −CO_2_H and −SO_3_H derivatized mica surfaces. In general, the formation of Au NC@CTAB SSs commences between 5 and 10 min after ethanol addition, depending on the supersaturation magnitude. At high Au NC concentrations (5.21 × 10^–7^ mol/L), the heterogeneous nucleation occurs earlier and at higher rates. On the contrary, at concentrations below 1.50 × 10^–7^ mol/L, heterogeneous nucleation was observed only in very few cases and with negligible nucleation rates. (Figure S4 and Table S4). The observed variation of ln(*J*_*n*_) as a function of 1/σ^2^ was found to follow a linear relationship (eq 5), which eventually allowed us to determine the interfacial energies (from eq 6) for the heterogeneous nucleation of Au NC@CTAB SSs on surfaces with −CO_2_H and −SO_3_Na groups (Figure 4, Tables 1 and S4).

**Table 1:** Kinetic and Thermodynamic Parameters^a^

			Δ*G*_*n*_ [*k*_B_*T*]		Thermodynamic term [*f*Ω^2^/(*k*_B_*T*)^3^]α^3^(1/σ^2^)		Kinetic term (*A*e^–*E*_A_/*k*_B_*T*^)
system	θ	α·10^–6^ [J/m^2^]	σ = 1	σ = 5	σ = 1	σ = 5	
CTAB −SO_3_Na	60	9 ± 1	3 ± 1	0.12 ± 0.04	4.5 ± 2	0.18 ± 0.08	16.7 ± 0.3
	90	6 ± 1	4 ± 2	0.16 ± 0.08			
	120	5 ± 1	4 ± 2	0.16 ± 0.09			
CTAB–CO_2_H	60	4 ± 4	1 ± 2	0.04 ± 0.06	0.6 ± 3	0.02 ± 0.12	16 ± 1
	90	3 ± 3	1 ± 2	0.05 ± 0.08			
	120	2 ± 2	1 ± 1	0.03 ± 0.04			
PAA–NH_2_	60	12.1 ± 0.1	10 ± 2	0.39 ± 0.01	10 ± 2	0.4 ± 0.08	18.5 ± 0.6
	90	8.2 ± 0.1	10 ± 0.2	0.41 ± 0.07			
	120	7 ± 0.5	10.5 ± 0.2	0.42 ± 0.09			

a The effective interfacial energy range for form factors from 60° to 90° and 120° contact angles, the nucleation barrier, and the kinetic and thermodynamic terms are given for each analyzed particle–substrate system.

Depending on the contact angle used to calculate the form factor, the magnitude of α varied from 5 × 10^–6^ to 9 × 10^–6^ J/m^2^ and 2 × 10^–6^ to 4 × 10^–6^ J/m^2^ for the substrates with −SO_3_Na and −CO_2_H moieties, respectively (Tables 1 and S4). These results suggest that the interaction between Au NC@CTAB and −SO_3_Na is less thermodynamically favorable than in the case of Au NC@CTAB and −CO_2_H, as reflected in the retrieved Δ*G*_*n*_ (eq 3) which, depending on the supersaturation (i.e., from 1 to 5) and contact angle (i.e., from 60° to 120°), ranged from 0.12 to 4 *k*_B_*T* for the −SO_3_Na and from 0.03 to 1 *k*_B_*T* for the −CO_2_H derivatized mica substrates (Table 1). Interestingly, we found that the former system is more favored from a kinetic point of view, as determined from eq 5 and the variation of ln(*J*_*n*_) as a function of 1/σ_2_ : 16.72 and 15.86 for −SO_3_Na and −CO_2_H, respectively (Table 1). However, it is important to note that the fitting quality of the experimental data of the CO_2_H derivatized mica substrates is low, and therefore the data cannot be treated as reliable as that of the −SO_3_Na derivatized mica substrates.

These results could thus explain the marked decrease in the extinction at 523 nm observed for the CTAB–SO_3_Na system during the kinetic studies performed with UV–vis–NIR spectroscopy (Figure 2) and a lower amount of Au NC@CTAB remaining in solution at the end of the heterogeneous nucleation experiment. It is worth noting that the kinetic term is greater than the thermodynamic one (i.e., for ions and molecules, the thermodynamics of the system generally dominate the nucleation behavior). This phenomenon could be explained by the lower effective interfacial energy and nucleation barrier in nanoparticle systems compared to atomic and molecular ones. For instance, the effective interfacial energy for the heterogeneous nucleation of calcium carbonate is between 7.2 × 10^–2^ and 9.5 × 10^–2^ J/m^2^, and the energy barrier ranges from 19 to 27 *k*_B_*T*.^68,69,71−73^ Moreover, the diffusion constants of nanoparticles are several orders of magnitude lower than that of ions and molecules, which could explain their high kinetic term values.

Finally, we evaluated the potential of the methodology described in this work to investigate the heterogeneous nucleation of superstructures constituted by Au NCs with different surface chemistry. Thus, we functionalized AuNCs with thiolated poly(acrylic acid) (AuNC@PAA) and determined α and Δ*G*_*n*_ following the approach utilized for Au NC@CTAB (Figures S5 and 6 and Table S5). In this case, the heterogeneous nucleation was triggered by adding CaCl_2_ using −NH_2_ derivatized mica substrates (i.e., due to electrostatic interactions between PAA and −NH_2_). Under these experimental conditions, the values of α and Δ*G*_*n*_ were found to range between 7 × 10^–6^ and 12.1 × 10^–6^ J/m^2^ and 0.39 and 10.5 *k*_B_*T*, respectively (Tables 1 and S5). These values are well above those noticed for the Au NC@CTAB system. However, the retrieved kinetic term was also higher, explaining the observed high nucleation rate at high supersaturation values (Figure 4).

## Conclusion

In summary, we have studied the phenomenon of heterogeneous nucleation using an Au nanoparticle-based model system. Particular emphasis has been placed on understanding the role of the chemical substrate surface nature onto such a process. Au NCs functionalized with cationic surfactant CTAB were synthesized in the first stage, and optimal conditions for their controlled self-assembly into Au NC@CTAB SSs were investigated. Then, we took advantage of the changes in the optical bands of Au NCs occurring during self-assembly to monitor the Au NC@CTAB SSs nucleation promoted at surfaces. In a second stage, the surface nature of quartz cuvettes was modified with organosilanes bearing distinct moieties: −CO_2_H, −SO_3_Na, −NH_2_, −OH, −CH_3_, and −CF_3_. This strategy allows us to investigate the role of substrate surface properties on the heterogeneous nucleation of Au NC@CTAB SSs. The highest extent of superstructure formation were observed for quartz surfaces with −CO_2_H and −SO_3_Na due to attractive electrostatic interaction with the positively charged Au NC@CTAB. Similarly, SEM characterization of mica substrates derivatized with −CO_2_H and −SO_3_Na groups demonstrated the most remarkable ability to efficiently stimulate the nucleation of the large superstructures. A combination of light microscopy and UV–vis–NIR spectroscopy was then utilized to gain insight into the thermodynamics of the Au NC@CTAB SSs heterogeneous nucleation process. Importantly, the use of CNT to evaluate the experimental data revealed that the interfacial energies and the nucleation barriers of the investigated nanoparticle systems are significantly lower than those of atoms and molecules where CNT can also be used for the evaluation of the heterogeneous nucleation experimental data. Moreover, the kinetic component plays a more significant role than the thermodynamic one in the heterogeneous nucleation behavior on Au NC@CTAB SSs, most probably due to the lower diffusion constants of nanoparticles. Notably, the reported methodology can be successfully applied to investigate Au NCs functionalized with PAA. This fact demonstrates the potential of using nanoparticle systems with different physicochemical features to investigate heterogeneous nucleation phenomena—a strategy that should eventually serve to study and unveil the mechanism behind complex nucleation processes observed in nature.

## Abbreviations

UV–vis: ultraviolet–visible
NIR: near-infrared
TEM: transmission electron microscopy
CTAB: cetyltrimethylammonium bromide
PEG: poly(ethylene glycol)
PAA: poly(acrylic acid)
SEM: scanning electron microscopy.
