# Supplementary material for: A Nanoparticle-Based Model System for the Study of Heterogeneous Nucleation Phenomena
Source: Langmuir. 2023 Mar 2;39(10):3580–8. doi: 10.1021/acs.langmuir.2c03034 (PMC10018769; doi:10.1021/acs.langmuir.2c03034)
Supplement: Supplementary file 1 — la2c03034_si_001.pdf [file la2c03034_si_001.pdf]

SUPPORTING INFORMATION

A Nanoparticle-Based Model System for the Study of Heterogeneous Nucleation Phenomena

Ann-Kathrin Göppert, Guillermo González-Rubio, Simon Schnitzlein, Helmut Cölfen**

Physical Chemistry, Department of Chemistry, University of Konstanz, Universitätsstraße 10, D-78465 Konstanz, Germany

TABLES

Table S1: Chemicals and incubation times used for substrate derivatization.

Chemical	Functional group	Coating time (h)
poly(sodium 4-styrenesulfonate)	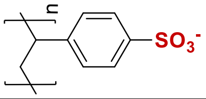	-
3-(triethoxysilyl)propyl-succinic anhydride	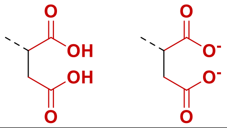	3
(3-aminopropyl)triethoxysilane	NH_2 NH_3^+	2
(3-acetoxypropyl)trimethoxysilane	OH	2
n-butyltriethoxysilane	CH_3	3
(3,3,3-trifluoropropyl)trimethoxysilane	CF_3	12
(Heptadecafluoro-1,1,2,2-tetrahydrodecyl)methyldichlorosilane	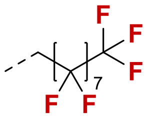	12

Table S2. Summary of the experimental conditions for nucleation experiments using Au NC@CTAB.

Experiment	Reaction vessel	Surface	EtOH content [vol.-%]	Surface/ volume ratio [1/cm]	Absorption at 523 nm [a.u.]
Homogeneous nucleation	Hydropho-bized cuvette	Quartz	38	-	1.5 - 1.7
Introducing surface	Micro test tube	Mica	31	0.39	1.5 - 1.7
Kinetics	Cuvette	Quartz	15	2.45	1.5 - 1.7
Light microscopy	Hydropho-bized cuvette	Mica	12	0.09	0.7 - 2.3

Table S3. Form factors *f* used to determine the α. For homogeneous and heterogeneous nucleation events, a spherical nucleus surrounded by the medium and a spherical nucleus attached to a substrate are considered, respectively. The geometry of the spherical nucleus on the substrate is defined by the contact angle, which influences the resulting form factor.

Nucleus	Angle [°]	*f*
Homogeneous nucleation	-	$(\frac{16}{3}\pi =)$ 16.76
Spherical – Heterogeneous nucleation	60	2.62
	90	$(\frac{8}{3}\pi =)$ 8.38
	120	14.14

Table S4. Determined nucleation rates, s, and the effective interfacial energy α for different form factors. Summarized results for the nucleation rates of the different particle surface combinations as a function of the Au NC@CTAB concentration (and the mean R^2 value for the straight lines fitted to the number of counted structures variation with time used to determine the nucleation rates). The slope (eq. 4) of fitted eq. 3 to the experimental variation of nucleation rate with $\frac{1}{\sigma^2}$ (and the corresponding mean R^2 value) used to determine the interfacial energy.

<table>
<tr><th>Moiety</th><th>[Au NC] 10^{-10} [mol/ L]</th><th>σ</th><th>J_n 10^6 [No/s·m²]</th><th>R^2</th><th>s $\frac{f\Omega^2}{(k_BT)^3}\alpha^3$</th><th>$R^2$</th><th>$f$</th><th>α [$10^{-6}$ J/m²]</th></tr>
<tr><td rowspan="5">-SO_3Na</td><td>1.50</td><td>1.44</td><td>2 ± 1</td><td>0.81</td><td rowspan="5">-4.5 ± 2</td><td rowspan="5">0.97</td><td rowspan="2">2.62</td><td rowspan="2">9 ± 1</td></tr>
<tr><td>2.04</td><td>1.96</td><td>6.0 ± 0.6</td><td>0.95</td></tr>
<tr><td>3.33</td><td>3.19</td><td>10.0 ± 0.1</td><td>0.85</td><td rowspan="2">8.38</td><td rowspan="2">6 ± 1</td></tr>
<tr><td>4.22</td><td>4.05</td><td>13 ± 5</td><td>0.99</td></tr>
<tr><td>5.21</td><td>5.00</td><td>18 ± 2</td><td>0.99</td><td>14.14</td><td>5 ± 1</td></tr>
<tr><td rowspan="4">-CO_2H</td><td>2.20</td><td>1.06</td><td>4 ± 3</td><td>0.92</td><td rowspan="4">-0.6 ± 3</td><td rowspan="4">0.54</td><td rowspan="2">2.62</td><td rowspan="2">4 ± 4</td></tr>
<tr><td>2.33</td><td>1.12</td><td>7 ± 4</td><td>0.94</td></tr>
<tr><td>3.37</td><td>1.62</td><td>6 ± 3</td><td>0.77</td><td>8.38</td><td>3 ± 3</td></tr>
<tr><td>3.71</td><td>1.79</td><td>6 ± 2</td><td>0.84</td><td>14.14</td><td>2 ± 2</td></tr>
</table>

Table S5. Calculation of the effective interfacial energy α for different form factors for Au NC@PAA.

<table>
<tr><th>Derivatization</th><th>s $\frac{f\Omega^2}{(k_BT)^3}\alpha^3$</th><th>$R^2$</th><th>Form factor f</th><th>Effective interfacial energy α [10^{-6} J/m²]</th></tr>
<tr><td rowspan="3">– NH_2</td><td rowspan="3">- 10 ± 2</td><td rowspan="3">0.89</td><td>2.62</td><td>12.1 ± 0.1</td></tr>
<tr><td>8.38</td><td>8.2 ± 0.5</td></tr>
<tr><td>14.14</td><td>7 ± 0.5</td></tr>
</table>

FIGURES

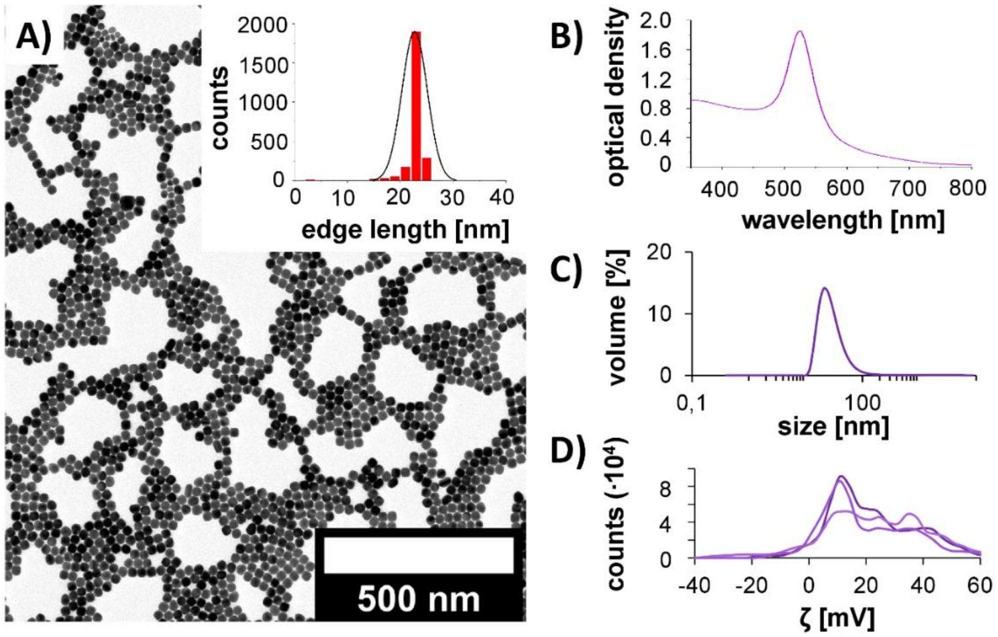

Figure S1. (A) TEM image, size distribution histogram (A inset), (B) UV-vis-NIR spectra, (C) DLS and (D) Zeta-potential measurements of the Au NC@CTAB used in this work.

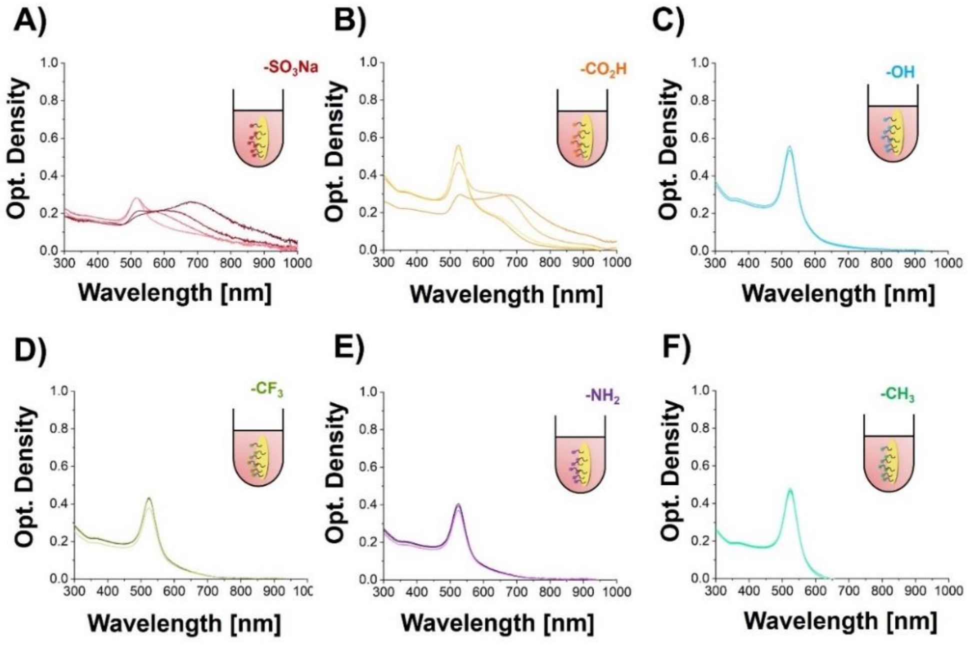

Figure S2. Heterogeneous nucleation of Au NC@CTAB SSs using derivatized mica substrates 15 min after nucleation initiation. UV-vis-NIR spectra of the Au@CTAB after 15 min of the addition of EtOH in the presence of different derivatized mica surfaces: (A) $-SO_3Na$, (B) $-CO_2H$, (C) $-OH$, (D) $-CF_3$, (E) $-NH_2$ and (F) $-CH_3$.

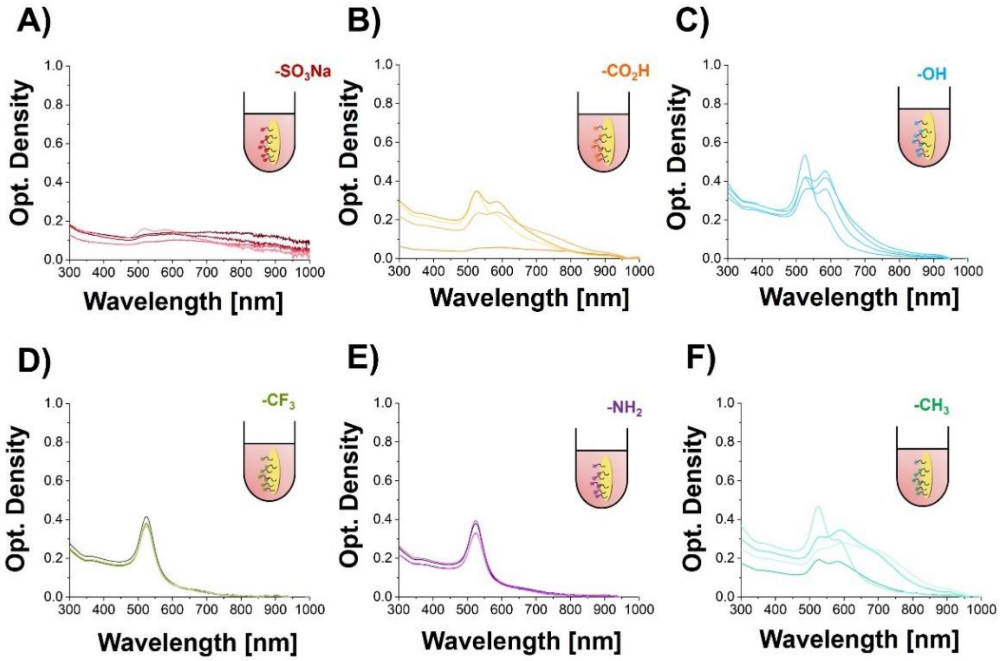

Figure S3. Heterogeneous nucleation of Au NC@CTAB SSs using derivatized mica substrates 24 h after nucleation initiation. UV-vis-NIR spectra of the Au@CTAB after 24 h of the addition of EtOH in the presence of different derivatized mica surfaces: ((A) −SO_3Na, (B) −CO_2H, (C) −OH, (D) −CF_3, (E) −NH_2 and (F) −CH_3.

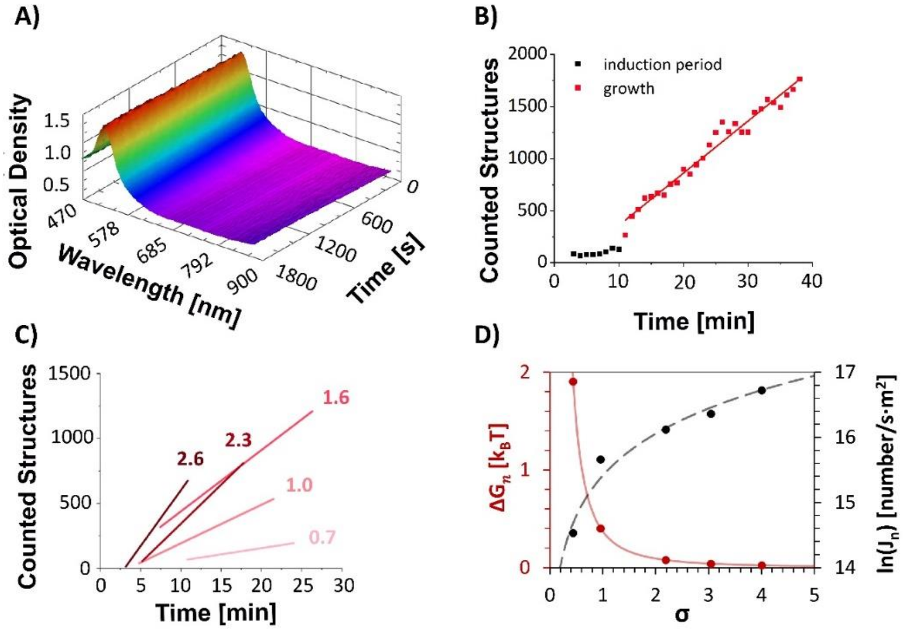

Figure S4. Determination of nucleation rate and energy barrier for the heterogeneous nucleation process of Au NC@CTAB SSs on −SO_3Na derivatized mica substrates. (A) The combination of UV-vis-NIR spectroscopy and (B) light microscopy revealed that nucleation and formation of Au NC@CTAB SSs only occurs on the sulphonate-derivatized mica substrate. (C) Depending on the supersaturation, it was possible to observe the formation of Au NC@CTAB SSs between 3 and 10 min after initiation of the nucleation process. The number of structures grow linearly with time, which allowed us to determine the nucleation rate of the heterogeneous nucleation process. (D) Variation of the nucleation rate and energy barrier as a function of the supersaturation. Increasing the supersaturation gave rise to a significant increase of the nucleation rate as a result of the nucleation barrier reduction.

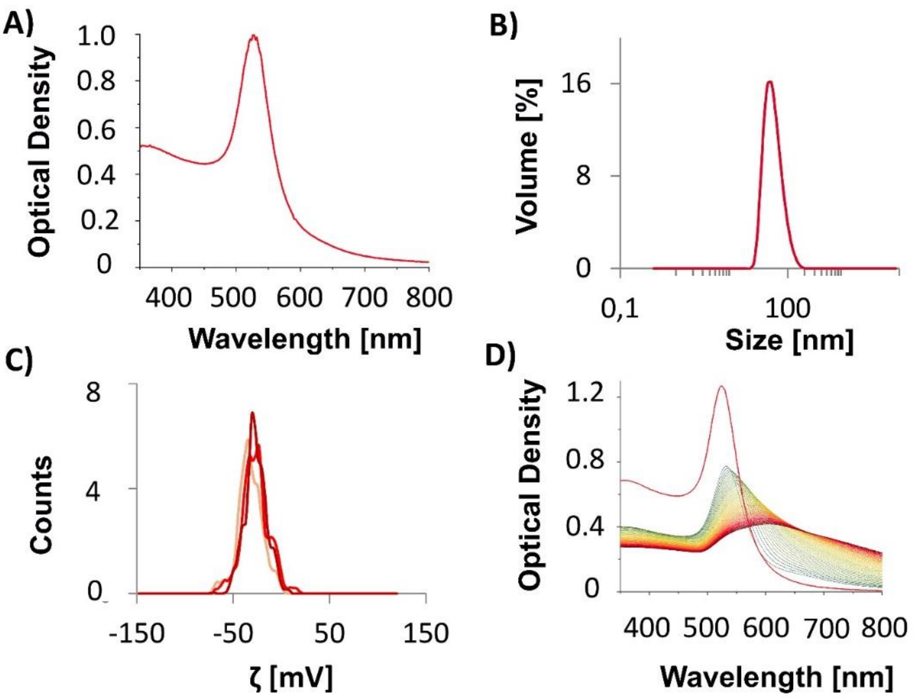

Figure S5. (A) UV-vis-NIR spectra, (B) DLS and (C) Zeta-potential measurements of the Au NC@PAA used in this work. (D) UV-vis-NIR spectra showing the LSPRs time evolution (over 75 min) of Au NC@PAA during colloidal destabilization process and subsequent formation of superstructures via homogeneous nucleation process

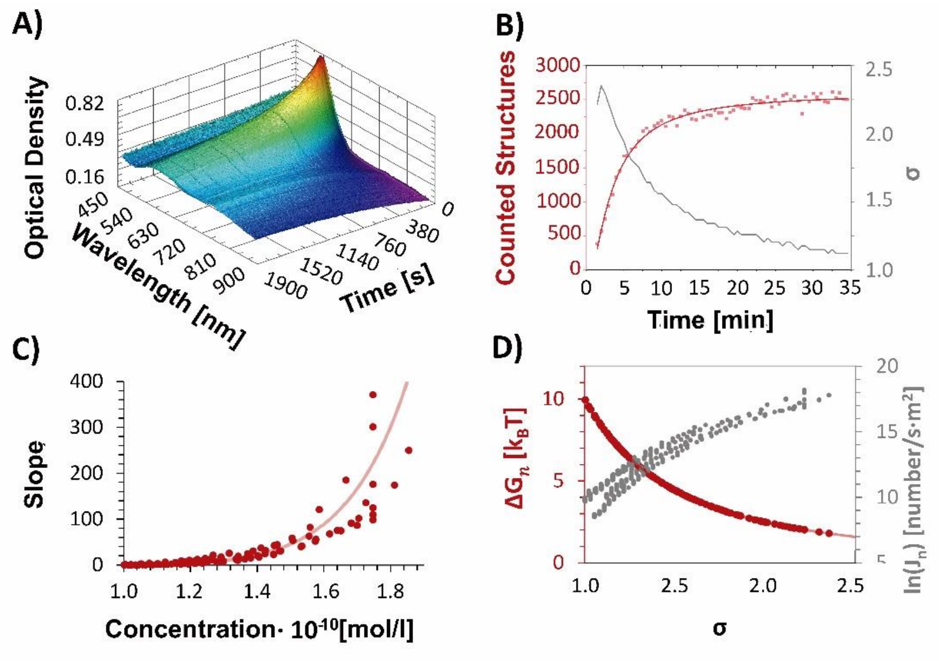

Figure S6. Determination of nucleation rate and energy barrier for heterogeneous nucleation process of Au NC@PAA SSs on $-NH_2$ derivatized mica substrates. (A) The combination of UV-vis-NIR spectroscopy and (B) light microscopy revealed that nucleation and formation of Au NC@PAA SSs occur on the $-NH_2$-derivatized mica substrates and in solution, most probably due to nuclei that are formed and detached from the mica surface. As a result, the supersaturation of Au NC@PAA decreases during the experiment in a logarithmic manner. (C) The slope of this fit at each time is the nucleation rate for the corresponding Au NC@PAA supersaturation value. (D) Variation of the nucleation rate (grey) and energy barrier (red) as a function of the supersaturation. Increasing the supersaturation gave rise to a significant increase of the nucleation rate as a result of the nucleation barrier reduction.
